# T cell-microglial interactions that impair myelin maintenance and regeneration: cellular mechanisms on white-matter associated changes

**DOI:** 10.3389/fimmu.2026.1813048

**Published:** 2026-05-29

**Authors:** Chaehyun Song, Yoonsung Lee, Man S. Kim

**Affiliations:** 1 Translational-Transdisciplinary Research Center, Clinical Research Institute, Kyung Hee University Hospital at Gangdong, College of Medicine, Kyung Hee University, Seoul, Republic of Korea; 2 Department of Medicine, Kyung Hee University College of Medicine, Seoul, Republic of Korea; 3 Center for Space Biomedical Science, NEXUS Institute, Kyung Hee University, Yongin-si, Republic of Korea

**Keywords:** CD8+ T cells, interferon-gamma, microglia, neuroinflammation, remyelination, white matter aging

## Abstract

White matter changes are a hallmark of aging and neurodegenerative diseases, characterized by progressive myelin deterioration and impaired regenerative capacities. Growing evidence indicates that maladaptive interactions between microglia and infiltrating T lymphocytes contribute to the progression of white matter changes. Recent single-cell and spatial transcriptomic studies have revealed aging-associated shifts in microglial and T cell states, offering new insights into how aging reshapes the immune-glial landscape. This review summarizes the cellular and molecular evidence across species, with emphasis on murine models, showing that aging alters microglial signaling profiles and promotes the recruitment of CD8^+^ T cells through cytokine signaling. We further examined how T cell-derived interferon-gamma induces transcriptional changes in microglia and oligodendrocyte lineage cells. The resulting interferon–responsive glial states destabilize myelin and limit remyelination, which are characteristic features of aging, multiple sclerosis, and neurodegeneration. By integrating molecular, genetic, and cellular insights, this review proposes a mechanistic framework using recent studies for how T cell–microglial interactions contribute to white matter alterations in aging and highlights the therapeutic opportunities to restore homeostasis and promote remyelination.

## 1 Introduction

White matter comprises nearly half of the human brain volume and plays an essential role in cognitive processing, with its integrity declining progressively with aging. Age-related white matter changes strongly correlate with cognitive decline, increased dementia risk, and motor dysfunction in elderly populations (1, 2). Despite their clinical significance, the cellular and molecular mechanisms driving white matter deterioration remain incompletely understood, limiting the development of effective therapeutic interventions.

Age-related white matter changes are detectable using Magnetic Resonance Imaging and are radiologically defined as White Matter Hyperintensities (WMH). Histopathological examination of WMH reveals diverse alterations, including myelin and axonal loss, alongside prominent vascular changes. While the development of WMH is widely accepted to arise from hypoxia and vascular disturbance, it is increasingly recognized as having a multifactorial etiology. Studies using animal models have demonstrated that the myelination and differentiation capacity of oligodendrocyte progenitor cells (OPCs) inherently declines with age (3, 4). Human primary oligodendrocyte lineage cells from pediatric groups demonstrated enhanced myelination of the nanofibers compared to those from adult groups, although the age group distinction remains dichotomous rather than a continuous spectrum (5). In a microstructural standpoint, dysregulated lipid metabolism causing aberrant cholesterol deposition, and ultrastructural disintegration of the myelin sheath further compromise white matter integrity (6, 7). The identification of immunologically active microglia near WMH lesions also suggests underlying glial pathologies (8).

This focus on glial pathologies is critical, as emerging evidence, mainly from animal models, has shown that white matter aging involves complex neuroimmune interactions (9). Two key cellular players have emerged as central orchestrators of this pathology: resident microglia and infiltrating T lymphocytes. Aging microglia undergo profound transcriptional and functional changes, transitioning from homeostatic myelin maintenance to dysfunctional, proinflammatory states (10). Paralleling these microglial changes is a marked influx of T lymphocytes into the aging white matter, with human brain data revealing a predominantly CD8^+^ population, suggesting a specific role for these cells in age-related white matter pathology (11, 12).

**Figure 1:**
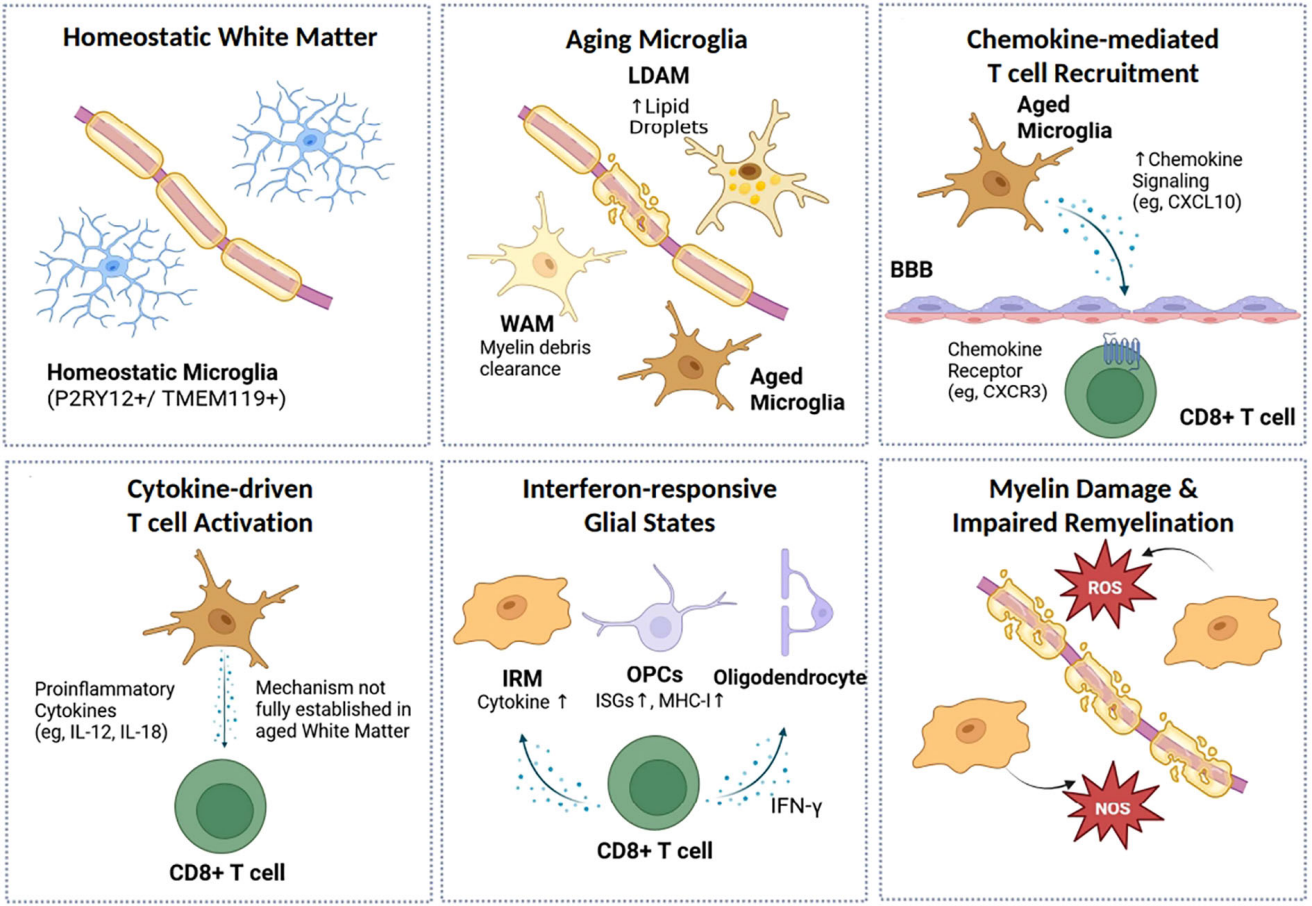
Overview of the immune-glial axis in white matter aging. (1) In healthy white matter, homeostatic P2RY12^+^ TMEM119^+^ microglia support myelin maintenance and few CD8^+^ T cells are present. (2) With aging, microglia adopt white matter–associated (WAM) and lipid droplet–accumulating (LDAM) states characterized by myelin debris handling and lysosomal stress. (3) Aging microglia and astrocytes upregulate chemokines such as CXCL10, which attract CXCR3^+^ CD8^+^ T cells across the blood–brain barrier. (4) Within the parenchyma, microglia-derived IL-12, IL-18, and type I interferons promote effector differentiation and IFN-γ production by CD8^+^ T cells. (5) T cell–derived IFN-γ reprograms microglia and oligodendrocyte lineage cells into IFN-responsive states enriched for interferon-stimulated genes, major histocompatibility complex (MHC) -I/II, and antigen-presentation machinery. (6) These reactive glial states drive oxidative stress, oligodendrocyte loss, and failure of remyelination, contributing to progressive white matter degeneration in aging. Created in BioRender. Song, C. (2026) https://BioRender.com/p6jfgxq. WAM, white matter-associated microglia; LDAM, lipid-droplet-accumulating microglia; BBB, blood–brain barrier, IRM, interferon-responsive microglia; OPCs, oligodendrocyte progenitor cells; ISGs, interferon stimulated genes; MHC, major histocompatibility complex; ROS, reactive oxygen species.

The mechanistic links connecting these cellular changes during aging are being increasingly elucidated. Dysfunctional microglia actively recruit CD8^+^ T cells through proinflammatory signals, such as chemokine signaling (13). Once present, CD8^+^ T cells establish bidirectional communication with glia through interferon-gamma (IFN-γ) signaling, inducing stress states in microglia and oligodendrocyte lineage cells (OLCs) that perpetuate inflammation and block regeneration (14–16). This creates a self-reinforcing cycle of immune dysregulation, myelin damage, and unsuccessful remyelination (**Figure 1**).

This review examines T cell-microglial interactions in white matter aging, focusing on T cell recruitment pathways, interferon-mediated crosstalk, and subsequent oligodendrocyte pathology. Drawing primarily on murine aging models and complementary human evidence collectively reviewed in Section 8, we evaluate evidence from aging and neurodegenerative models, discuss targeted therapeutic strategies, and identify future research priorities.

## 2 Current understanding of genetics associated with white matter changes

Large-scale Genome-Wide Association Studies (GWAS) have been pivotal in elucidating the genetic architecture of white matter pathology. GWAS of over 43,000 Diffusion MRI scans identified hundreds of variants across more than 150 genomic regions that dictate inter-individual differences in white matter microstructure (17). The genetic effects observed were strikingly enriched in glial regulatory elements in oligodendrocytes, microglia, and astrocytes, rather than neurons. Significantly, many implicated loci overlapped with established risk genes for major brain disorders, establishing a strong genetic linkage between white matter integrity and brain disease susceptibility.

Subsequent GWAS focused on pathologic WMH identified 20 distinct genome-wide significant loci, demonstrating selective enrichment in genes expressed by vascular cell types as well as astrocytes and oligodendrocytes (18). These findings suggest an integrated mechanistic model that white matter aging stems from a coordinated multi-cellular deterioration. The specific mechanism of how these genetic variations lead to pathology remains to be elucidated. However, these findings provide genetic support for the centrality of glial cells, particularly microglia and oligodendrocytes, in white matter pathology, reinforcing the mechanistic framework explored in this review, wherein dysregulated immune-glial interactions drive white matter deterioration.

## 3 Microglia: key players of myelin maintenance

### 3.1 Physiological functions of microglia in white matter

Microglial states are highly diverse, reflecting the pathophysiological contexts (**Table 1**). While the inherent plasticity of microglia defies strict categorization, the advent of RNA sequencing provides a framework for defining functional states through transcriptomic signatures. It is important to note that these transcriptomic signatures are not exclusive to the pathological contexts after which they are named and overlapping profiles have been identified across distinct biological settings (26). Named subpopulations such as those listed in **Table 1** are therefore used as descriptive conveniences to aid interpretation, rather than as fixed or mutually exclusive categories (27).

**Table 1:** Comparative analysis of representative microglial transcriptomic signature in homeostasis, aging, and disease.

Microglial Transcriptomic Signature	Context	Key DEGs	Enriched pathways	Primary function	Model	References
Homeostatic Microglia	Physiologic Conditions	*P2RY12, CX3CR1*	Positive regulation of GTPase pathway, Regulation of GTPase activity	Surveillance, Debris clearance, Trophic support	Mouse, Human	(19–22)
WAM	Aging	*Apoe, Cst7, Bm2, Lyz2, Cd63, Clec7a, Ctsb, Ctss, Ctsz, H2-D1, H2-K1*	Hypoxia-inducible factor signaling, Glycolysis/gluconeogenesis, Lysosome	Myelin debris phagocytosis, Lipid metabolism	Mouse	(23)
DAM	Neurodegenerative disease, Development, Aging	*Trem2, Apoe, Ctsd, Lpl, Tyrobp,Cd9, Itgax (Cd11c), Clec7a, Cd63*	Lysosome, Positive Regulation of immune system, Response to wounding	Disease-associated phagocytosis	Mouse, Human*	(24)
LDAM	Aging	*Cd63, Atp6v1a, Atp6v1c1, Atp6v1g1, Tuba1, Rab5b, Rab7, Cd22, Cat, Kl, Ppp1cb, Jak, Rap1b, Plin3*	Negative regulation of protein metabolic process, Positive regulation of cholesterol storage, Positive regulation of lipid storage	Impaired phagocytosis, ROS production	Mouse	(25)
IFN- responsive microglia	Aging and inflammatory conditions	*ISGs (Stat1, Ifit3, Usp18, Ifit27lea), Fcgr1, Cxcl10, Ccl12, Ccl2*	Response to IFN-alpha and beta, Lymphocyte chemotaxis, Immune response	ISG-high reactive microglial state, Immune response modulation	Mouse	(16)

DEGs, differentially expressed genes; WAM, white matter-associated microglia; LDAM, lipid-droplet-accumulating microglia; DAM, disease-associated microglia; ISGs, interferon-stimulated genes; ROS, reactive oxygen species.

* In humans, DAM are further divided into clusters enriched for inflammatory, lysosomal/lipid-metabolic, and ribosome-related pathways (22).

Throughout life, microglia perform essential homeostatic functions that maintain white matter health. These cells continuously survey their microenvironment, detecting and responding to subtle changes in tissue integrity (20). Through this dynamic surveillance, microglia actively support oligodendrocyte development and myelination by secreting trophic factors and phagocytosing excess OPCs (19, 21). Microglial phagocytic activity is essential for clearing myelin debris, which contains molecules that inhibit OPC differentiation and axonal regeneration (28). By efficiently removing this debris, microglia create a permissive environment essential for remyelination (29, 30).

Aging progressively compromises these functions, as increased myelin debris and lysosomal burden predispose microglia to dysregulated inflammation (31). This is evidenced by lipid droplet-accumulating microglia (LDAM) in aged mice, which exhibit impaired phagocytosis alongside elevated reactive oxygen species (ROS) and proinflammatory cytokine production (25). This transition from protective clearance to a maladaptive state drives white matter degeneration and cognitive decline, with similar patterns observed in non-human primates (32).

### 3.2 Genetics of age-related changes in microglia

During aging, microglia undergo profound transcriptional changes and a selective erosion of homeostatic machinery across species, compromising their functional integrity. A single-cell RNA sequencing study in mice identified microglial subpopulations that proliferate with aging, characterized by the increased expression of inflammatory mediators and interferon-response genes (ISGs) (10). This upregulation is notable because IFN-stimulated transcriptional states in glia have been implicated to suppress homeostatic functions and impede remyelination.

In the aging mouse white matter, these transcriptional changes culminate in a specialized subpopulation termed White Matter-Associated Microglia (WAM). WAM share a partially overlapping gene profile with disease-associated microglia, a subset of microglia observed in neurodegenerative diseases such as Alzheimer’s disease (AD) (24, 33). The overlapping genetic signatures include the upregulation of phagocytosis and lipid-handling metabolism (23). These features enable WAMs to clear myelin debris and maintain white matter homeostasis, though this protective capacity becomes overwhelmed with advanced age (34). Despite these significant mechanistic insights gained largely from murine models, a research gap remains in translating these findings to human pathology.

Comparative analyses of aged human microglia across species reveal a conserved loss of P2RY13 and a critical downregulation of P2RY12 in the human brain, both purinergic sensory machineries essential for rapid process extension and surveillance (35–37). This erosion of sensory machinery is accompanied by broader homeostatic failure, where transcriptional changes often represent inadequate compensatory efforts. For instance, SLC16A3 upregulation in aged microglia does not fully restore metabolic capacity (37), suggesting incomplete compensatory responses.

## 4 CD8+ T cell recruitment to aging white matter

### 4.1 T cells in the brain

The presence of T cells in the central nervous system (CNS) has historically been considered limited under physiological conditions due to the restrictive nature of the blood–brain barrier. However, recent studies have demonstrated that small populations of T cells, including CD8+ T cells, can be detected in postmortem human brain tissue, particularly in the perivascular spaces and cerebrospinal fluid (CSF) (38, 39). These cells closely resemble tissue-resident memory T cells, characterized by the high expression of CD69 and tissue-homing chemokine receptors.

This homeostatic quiescence, however, can be progressively disrupted by age-related neuroimmune remodeling. Spatial transcriptomics in mice reveal a robust accumulation of T cells specifically within white matter tracts during aging (40). Although such massive expansion in aging human white matter remains to be definitively proven, the frequent detection of activated, antigen-experienced T cells in the CSF of patients with neurodegeneration (41, 42) suggests a latent potential for these cells to transition into a reactive state.

Once activated or recruited in excess, these CD8+T cells adopt a pronounced cytotoxic effector phenotype. In aged mouse models, these infiltrating cells express high levels of granzymes and IFN-γ (16, 43). Evidence from oligodendrocyte-mutant mice further confirms that such CNS-resident CD8+ T cells utilize perforin and granzyme B to directly mediate oligodendrocyte injury and axonal loss (44), establishing a mechanistic link between age-related T-cell infiltration and white matter degeneration.

### 4.2 CXCL10–CXCR3 axis: an emerging mechanism for T cell recruitment to aging white matter

The CXCL10–CXCR3 axis has emerged as a candidate mechanism for T-cell recruitment to the aging CNS. Study in mouse models has shown that aging-associated microglia and astrocytes near the optic nerve upregulate CXCL10, a chemokine that binds CXCR3 on T cells and directs their migration to the sites of inflammation. In this model, genetic deletion or bone marrow chimera experiments of CXCL10 or CXCR3 reduces CD8+ T cell accumulation in aged white matter, mitigates myelin abnormalities and prevents axonal degeneration (13).

Complementing these findings, human‐relevant AD organoid studies have shown that CXCL10–CXCR3 signaling similarly governs T cell recruitment and associated neural injury. Elevated CXCL10 levels within glial-rich compartments attracted CXCR3^+^ CD8^+^ T cells, and CXCR3 neutralization reduced T cell infiltration and attenuated inflammatory neurotoxicity (45). Although derived from an AD context rather than aging white matter, these results provide convergent evidence that CXCL10–CXCR3-dependent chemotaxis can operate in human-derived CNS environments.

The CXCL10–CXCR3 signaling axis has been well characterized in peripheral inflammatory and autoimmune disorders (46). In the context of neuroinflammation, CXCR3 has been implicated in multiple sclerosis (MS), where it is expressed within inflammatory brain lesions and is enriched in CD4+ T cells in the CSF (47). Reactivation of this pathway in aging white matter is consistent with a model in which microglial dysfunction is linked with adaptive immune dysregulation and subsequent myelin pathology.

### 4.3 T cell pathology in the CNS beyond normal aging

The pathogenic role of T cells extends beyond aging and is increasingly recognized in white matter-related diseases. In AD murine models and human brain tissues, CD8^+^ T cells are frequently found adjacent to MHC-II^+^ and IFN-responsive glial cells (48). A similar phenomenon has been observed in a mouse model of CAR T cell therapy, where systemic T cell activation induces sustained microglial reactivity, subsequent oligodendrocyte loss, and cognitive deficits (49). Together, these observations suggest that T cell-driven inflammatory signaling can converge on vulnerable white matter glial states across diverse contexts.

## 5 IFN-responsive glial states in aging white matter

A growing body of evidence indicates that CD8^+^ T cells orchestrate a coordinated reactive program across multiple glial populations in the aging white matter. These glial populations share a common ISG signature (Stat1, Ifit3, Usp18, and Ifi27l2) and stress response pathways, in addition to other cell type-specific responses (16).

### 5.1 IFN-responsive microglia

IFN-γ induces a reactive microglial state characterized by the upregulation of MHC-II molecules and increased production of proinflammatory cytokines, including tumor necrosis factor-α, interleukin-6, ROS, and nitric oxide synthase (NOS) (50–52).

Functional evidence from neurodegenerative disease models supports the pathogenic relevance of microglial MHC-II upregulation. In murine models of Parkinson’s disease, microglial MHC-II induction was required for antigen presentation, CD4 T cell proliferation, and dopaminergic neurodegeneration, with MHCII knockout preventing this cascade (53). Similarly, in AD models, MHC-II-expressing microglia facilitated T cell-mediated responses, demonstrating that microglial antigen presentation can orchestrate adaptive immune activation across diverse neurodegenerative contexts (54).

Increased oxidative and nitrosative stress also poses a major threat to oligodendrocytes. NOS induction produces excessive nitric oxide, which forms peroxynitrite, mediating oxidative injury and oligodendrocyte toxicity (55). *In vitro* studies using human primary oligodendrocytes have further demonstrated that oxidative stress induces oligodendrocyte apoptosis (56).

### 5.2 IFN-responsive oligodendrocytes

OLCs are critical targets of IFN-γ signaling. The effects of IFN-γ have long been studied in the context of MS, exhibiting dose-dependent outcomes. High concentrations of IFN-γ impair oligodendrocyte survival and function, leading to demyelination. In contrast, sub-apoptotic IFN-γ levels can initially induce a protective integrated stress response (ISR) through endoplasmic reticulum (ER) stress pathways (57). Although IFN-γ triggers ISR activation in oligodendrocytes, stress adaptation remains incomplete, and prolonged IFN-γ exposure ultimately compromises oligodendrocyte survival and function (58).

In addition to these stress-related effects, IFN-γ reshapes the immune properties of OLCs. IFN-γ drives OPCs to upregulate antigen-presentation machinery, including MHC class I molecules and the immunoproteasome subunit PSMB8 (59, 60). Through this shift, OPCs gain the capacity to present myelin-derived antigens and potentially activate myelin-reactive CD8^+^ T cells while simultaneously becoming vulnerable to cytotoxic targeting. Similarly, single-cell transcriptomics of aged mouse white matter identified analogous IFN-responsive OLC states, enriched for ISGs and MHC-I components, where genetic depletion of lymphocytes markedly reduced these populations and prevented age-related oligodendrocyte loss (16). Altogether, these features point toward a bidirectional mode of interaction between OLCs and T cells.

## 6 Cellular and molecular mechanisms of remyelination failure

### 6.1 Cytokine-mediated inhibition of OPC differentiation

The capacity for remyelination declines markedly with advancing age, a phenomenon observed in non-human primates and human OLCs (4, 5, 61). A major explanation for these age-driven changes is the reduced differentiation potential of OPCs into mature, myelinating oligodendrocytes during aging (62).

Elevated cytokines, particularly IFN-γ, create an inflammatory barrier to OPC maturation. While IFN-γ can inhibit OPC cell-cycle exit and differentiation *in vitro* (63), sustained *in vivo* exposure in mice triggers ER stress, impairing oligodendrocyte survival and blocking remyelination (14). To overcome this blockade, recent therapeutic strategies have targeted these specific pathways. In a mouse model with ectopic IFN-γ expression, treatment with Sephin1, which prolongs the adaptive phase of the integrated stress response, together with bazedoxifene, a pro-differentiation agent that counteracts the maturation block, markedly enhanced remyelination (64).

### 6.2 Myelin debris accumulation and remyelination inhibition

Inefficient clearance of myelin debris is another critical mechanism linking immune dysfunction with impaired remyelination and axonal regeneration. Myelin contains several inhibitory molecules including myelin-associated glycoprotein, Nogo-A, and oligodendrocyte-myelin glycoprotein that signal through NgR1 and PirB receptors to impede OPC differentiation and axonal regeneration (65–67).

Several phagocytic receptors and integrins have been identified as mediators of myelin phagocytosis, including CD22, TAM family receptors (Tyro3, Axl, MerTK), and TREM2, primarily through neurodegenerative disease animal models (68–70). CD22, an inhibitory receptor upregulated in aged microglia in murine models, has been shown to suppress phagocytic capacity including myelin debris clearance (69). Preliminary human evidence suggests CD22 expression in microglia-like cells derived from monocytes, though validation in brain tissue microglia remains needed (71). TAM family receptors play a key role in phagocytic clearance upon apoptosis, activated upon binding of Pros1 and Gas6. Axl and MerTK have shown particular relevance to microglia (72).

TREM2 is a plasma membrane receptor present on the microglial surface, promoting cell activation such as phagocytosis and cell motility through different signaling pathways upon ligand binding (73). TREM2 supports microglial proliferation, myelin phagocytosis, and myelin regeneration in aging murine models (23, 68, 74). However, transcriptomic and flow cytometric studies report decreased surface TREM2 expression in aged mice, accompanied by impaired amyloid beta phagocytosis *in vitro* (75, 76), suggesting age-dependent functional decline. The degree of TREM2 expression has been shown to directly impact phagocytic function in microglia (77), which is consistent with the failure of a TREM2-targeting antibody in a recent phase 2 clinical trial in AD patients, potentially reflecting limited therapeutic target availability due to age-dependent surface TREM2 decline (78). Together, these findings suggest a progressive decline in microglial phagocytic capacity with aging, with potential consequences for myelin debris clearance and remyelination.

### 6.3 Genetics and epigenetics behind impeded remyelination

A single-cell genomic study has revealed that oligodendrocytes accumulate somatic mutations at a significantly faster rate than neurons during aging, with oligodendrocytes acquiring single nucleotide variants 81% faster than neurons in human brains (79). These mutations exhibit mutational signatures overlapping with those observed in glial tumors and are preferentially enriched in inactive genomic regions marked by suppressive epigenetic regulators such as H3K9me3. The functional implications of these somatic mutations remain to be elucidated.

In addition to somatic mutations, converging evidence from animal models and human MS studies suggest that epigenetic dysregulation contributes to impaired OPC differentiation and remyelination in aging, though the specific histone marks and mechanisms involved remain incompletely characterized across model systems. In murine aging models, age-dependent decline in histone deacetylases (HDACs) recruitment to differentiation inhibitor promoters impairs remyelination efficiency (80). In human MS contexts, broad shifts in activating and repressive histone marks have been observed, suggesting epigenetic silencing of myelination programs (81). Directly converted oligodendrocytes from fibroblasts of MS donors across age groups displayed reduced H3K9me3 with advancing donor age, reflecting epigenetic instability associated with cellular senescence (82). Still, epigenomic characterization of normally aged human oligodendrocytes remains an important gap in the field, with most evidence derived from murine models or disease contexts.

The cumulative impact of these genetic and epigenetic alterations often manifests as cellular senescence, a state that further compromises the regenerative potential of the OPC pool. Aging-associated senescent OPCs in mice display elevated expression of Serpina3n, a marker of disease-associated oligodendrocytes previously linked to neuroinflammatory responses; Nol3, an anti-apoptotic protein; and Skap2, an integrin signaling adapter involved in cell adhesion and migration (83). The functional impact of these changes remains to be fully clarified, yet such senescent OPCs may render the local glial environment less supportive of efficient remyelination.

## 7 Therapeutic implications and future directions

Targeting the components of the pathological immune–glial microenvironment that emerges during white matter aging may provide a conceptual basis for novel therapeutic strategies.

### 7.1 Modulating interferon signaling

Given the central role of IFN signaling in mediating T cell-microglial-oligodendrocyte crosstalk, this pathway represents an attractive therapeutic target. The JAK-STAT pathway plays a central role in transmitting IFN-γ and other cytokine signals in glial cells, and clinically approved JAK inhibitors may suppress pathogenic IFN-responsive glial states. For example, in 5xFAD transgenic AD mouse brain slices, baricitinib, a JAK-STAT pathway inhibitor, prevented myelin damage induced by microglia and reduced IFN-γ-mediated MHC-II induction (48).

However, careful titration is necessary because complete blockade of the IFN pathway through JAK inhibitors can impair antimicrobial immunity (84). Alternatively, targeting specific interferon regulatory factors or ISGs most relevant to glial pathology, while preserving broader antiviral responses, may provide a more nuanced therapeutic approach (85). Enhancing the protective functions of microglia while suppressing their pathogenic inflammatory activities represents another promising strategy (86).

### 7.2 Promoting oligodendrocyte resilience and regeneration

Enhancing the intrinsic capacity of OLCs to survive inflammatory stress and differentiate effectively represents a complementary therapeutic approach (87). Pharmacological agents promoting OPC differentiation, including bazedoxifene and metformin, show promise in preclinical models (88, 89). However, these agents may be insufficient in highly inflammatory environments, where cytokine-mediated differentiation blockade overwhelms pro-differentiation signals. In such cases, combination therapies that simultaneously suppress inflammation and actively promote OPC differentiation may provide synergistic benefits (64).

Age-specific interventions may be necessary, as aged OPCs in aging mouse models have displayed distinct proteomic and metabolic profiles compared with their younger counterparts (61, 90). Targeting age-related declines in chromatin regulation, activity-dependent signaling, and metabolic homeostasis could help rejuvenate aged OPCs and restore their differentiation potential (90–92), particularly relevant for preventing or treating age-related white matter degeneration.

Specifically, as described in Section 6.3, epigenetic dysregulation of oligodendrocyte lineage cells represents a key contributor to remyelination failure, offering a promising therapeutic avenue. ESI1 (Epigenetic-silencing-inhibitor 1), a small molecule initially discovered as an HDAC3 inhibitor, was recently reported to increase myelin gene expression in human oligodendrocyte organoids and promote oligodendrocyte maturation in aged murine models (81). Mechanistically, ESI1 inhibits HDAC3-mediated silencing of myelination genes while simultaneously increasing activating histone marks such as H3K27ac and reducing repressive marks including H3K27me3 and H3K9me3, thereby effectively reversing the epigenetic state associated with remyelination failure in aging.

### 7.3 Targeting the CXCL10–CXCR3 axis

The identification of the CXCL10–CXCR3 axis as a mediator of pathogenic cytotoxic T cell recruitment to the white matter highlights this pathway as a potential therapeutic target. In aging white matter models of mouse, genetic deletion of CXCL10 and bone marrow chimera experiments of CXCR3 reduces CD8+ T cell accumulation and ameliorates myelin pathology, demonstrating proof of concept for this approach (13).

Small-molecule CXCR3 antagonists have already demonstrated efficacy in autoimmune models and are currently in clinical trials for various autoimmune diseases (93–95). However, successful translation to aging brain therapy must navigate significant hurdles as systemic inhibition may compromise essential immune surveillance against infections or tumors, and the inherent promiscuity of chemokine signaling complicates achieving precise target specificity (96, 97).

Altogether, these findings suggest that CXCL10–CXCR3 modulation may offer therapeutic potential, but successful translation will likely require strategies that balance effective immune regulation with the preservation of essential host-defense functions.

## 8 Current status and limitations of human evidence

Current human evidence for the immune-glial interactions described in this review remains fragmented, with most studies focusing on individual cell types in isolation. For microglia, transcriptomic profiling through single-cell and single-nucleus RNA sequencing has identified aging-associated changes in human brain tissue, though these studies lack a unified consensus, likely reflecting differences in tissue source, isolation protocols, and sample size (35, 98–101). For T cells, postmortem studies have confirmed the presence of CD8+-predominant tissue-resident memory T cells in subcortical white matter (12, 39), while CSF-based studies provide indirect evidence of immune dysregulation in aging and neurodegeneration (41, 42). Whether these T cells undergo aging-associated functional changes within white matter, and how they interact with resident glia, remains uncharacterized. Human white matter-specific oligodendrocyte transcriptomics remains largely absent, with available data predominantly derived from broader cortical studies designed to compare normal aging with AD, not designed to capture aging white matter pathology (102, 103).

These limitations collectively highlight two critical gaps: the absence of white matter-specific, aging-focused transcriptomic data across all three cell types, and the lack of any integrated human evidence capturing the full immune-glial interaction framework described in this review. Addressing these gaps will require white matter-targeted single-cell and spatial transcriptomic studies with adequate sample sizes. Complementary approaches, including multiplexed imaging to resolve cell-cell proximity and oligodendrocyte-immune co-culture or organoid systems for functional validation, will be essential to translate findings from murine models to human pathology.

## 9 Conclusion

Emerging research on white matter pathology highlights the complex interplay between maladaptive neuroimmune interactions. Successful therapeutic strategies will likely require multimodal approaches that simultaneously disrupt pathogenic T cell-microglial interactions, restore beneficial microglial functions, and actively promote oligodendrocyte resilience and regeneration. Crucially, the translation of findings from animal models to the clinic will require rigorous validation through human tissue studies. As we continue to unravel these cellular and molecular drivers, the expanding landscape of therapeutic targets offers a transformative path toward treating these historically intractable conditions.
